# Gynecologic Cancer InterGroup CA125 response has a high negative predictive value for CHK1 inhibitor RECIST response in recurrent ovarian cancer

**DOI:** 10.1038/s41598-024-68338-2

**Published:** 2024-07-29

**Authors:** Kristen R. Ibanez, Duncan Donohue, Tyler Malys, Jung-Min Lee

**Affiliations:** 1 Women’s Malignancies Branch, Center for Cancer Research, National Cancer Institute, National Institutes of Health, 10 Center Drive, Building 10, Room 6B12, Bethesda, MD 20892 USA; 2 Statistical Consulting and Scientific Programming Group, Computer and Statistical Services, Data Management Services, Inc. (a BRMI Company), NCI, Frederick, MD 21702 USA

**Keywords:** Ovarian cancer, Predictive markers, Cancer, Biomarkers, Medical research, Molecular medicine, Oncology

## Abstract

We investigated the association of CA125 response with prognosis and RECIST response/progressive disease (PD) criteria in recurrent high grade serous ovarian cancer (HGSOC) patients treated with a cell cycle checkpoint kinase 1 inhibitor (CHK1i), prexasertib. 81 patients had measurable disease per RECISTv1.1, of which 72 and 70 were measurable by Gynecologic Cancer InterGroup (GCIG) CA125 response and PD criteria, respectively. Univariate and multivariate analyses showed that GCIG CA125 response (n = 32) is associated with improved progression-free survival (PFS) and overall survival (OS) compared to no GCIG CA125 response (n = 40) (median PFS 8.0 vs. 3.5 months [HR: 0.30, 95% CI: 0.18–0.51, *p* < 0.0001]; median OS 19.8 vs. 10.0 months [HR: 0.38, 95% CI: 0.23–0.64, *p* < 0.001]) independent of BRCA mutation status, platinum-sensitivity, previous PARP inhibitor therapy, ECOG performance status, and FIGO stage. Notably, GCIG CA125 response had a high negative predictive value (NPV: 93%, 95% CI: 80–98), but poor positive predictive value (PPV: 53%, 95% CI: 35–71) in predicting RECIST response. CA125 PD criteria also showed poor concordance with RECIST PD (PPV 56%, 95% CI: 40–71; NPV 33%, 95% CI: 17–54). Therefore, serum CA125 may be useful as a highly accessible prognostic and predictive biomarker to CHK1i therapy in recurrent HGSOC.

## Introduction

Ovarian cancer is rare, but represents the most lethal gynecologic malignancy in the United States^1^, of which high grade serous ovarian cancer (HGSOC) is the most common (~ 75%) subtype^2^. Approximately two-thirds of patients with advanced-stage HGSOC experience relapse despite initial responses to cytoreductive surgery and platinum-based chemotherapy^3^. The prognosis for patients with recurrent disease remains poor with limited treatment options or readily accessible and reliable biomarkers^3^. Therefore, new strategies are needed.

One novel therapeutic approach for recurrent HGSOC is targeting cell cycle signaling blockade mediated by ataxia telangiectasia mutated- and Rad3-related (ATR) and cell cycle checkpoint kinase 1 (CHK1)^3,4^. Several ATR inhibitors such as berzosertib and camonsertib or CHK1 inhibitors (CHK1i) remain under clinical investigation for advanced solid tumors and ovarian carcinoma (e.g. NCT02627443, NCT04497116, NCT02808650)^5^. Prexasertib (a.k.a. ACR-368, LY2606368), a second generation CHK1i, has previously shown clinical activity and tolerability profile in subsets of relapsed platinum-resistant recurrent HGSOC^6–8^. Prexasertib is currently under investigation in a multi-center, registration-intent phase II clinical trial (NCT05548296) for patients with recurrent platinum-resistant HGSOC. While ATR/CHK1 pathway targeting therapies are promising, clinical biomarkers coming from a readily attainable source such as blood or urine, are not yet available to predict response or disease progression (PD) while on prexasertib therapy.

Serum cancer antigen 125 (CA125), a secreted form of the transmembrane mucin MUC16^9^, is a highly accessible biomarker which has shown utility for monitoring disease response and predicting long-term prognosis from initial platinum-based chemotherapy in epithelial ovarian cancer^10,11^. Gynecologic Cancer InterGroup CA125 criteria (GCIG CA125) have been widely used in clinical trials and practice for assessment of tumor response and progression^12^. However, there remains little evidence regarding its use as a biomarker for disease response and PD for small molecule inhibitors as well as for its relevance for heavily pretreated recurrent HGSOC. Hence, more studies are needed to identify the relationship of CA125 on response to targeted therapies in drug-resistant recurrent ovarian carcinoma.

Here, we performed a pooled analysis using data from 3 recurrent HGSOC cohorts of a single center phase II study of prexasertib (NCT02203513) to identify the association of CA125 response criteria with prognosis. We also compared longitudinal CA125 changes to Response Evaluation Criteria in Solid Tumors (RECIST) outcomes to elucidate the predictive value of CA125 for RECIST response and progression to prexasertib. Lastly, we examined whether there are subgroup differences according to platinum sensitivity, BRCA mutation status, prior use of poly(ADP-ribose) polymerase (PARP) inhibitors, International Federation of Gynecology and Obstetrics (FIGO) stage at diagnosis, and Eastern Cooperative Oncology Group (ECOG) performance status.

## Methods

### Study population

This post-hoc analysis examined data from an open-label, single-center phase II study (NCT02203513) evaluating women aged 18 years or older with measurable, recurrent high grade serous or high grade endometrioid ovarian carcinoma with no limit on the number of prior therapies. CA125 only disease without RECIST measurable tumors was not eligible. All participants provided written informed consent prior to enrollment. The study was approved by the Institutional Review Board of the Center for Cancer Research (CCR), NCI, USA. All procedures were carried out in accordance with the ethical principles founded in the Declaration of Helsinki and its amendments. The full study methodology for this trial has previously been published in detail^6–8^. Briefly, patients received intravenous prexasertib 105 mg/m^2^ once every two weeks until PD, unacceptable toxicity, or patient withdrawal of consent. Imaging for RECIST version 1.1 (v1.1) criteria was obtained at baseline and every two cycles and laboratory assessments including serum CA125 obtained at baseline and prior to administration of each cycle (every 4 weeks). The primary endpoint of the phase II study was investigator-assessed tumor response per protocol based on RECIST v1.1 in evaluable patients.

### Evaluation of disease response and progression by RECIST v1.1 and GCIG CA125 criteria

Disease response was determined by investigators using RECIST v1.1, with tumors measured by computed tomography (CT) or magnetic resonance imaging (MRI). Imaging was conducted every two cycles for the first four years, and every three cycles thereafter. Progression-free survival (PFS) was defined as the time from treatment onset until the date of radiographic progression or the treatment discontinuation, whichever occurred earlier. Overall survival (OS) was defined as time from treatment onset until death or censored when data was locked on March 19, 2023.

CA125 was assessed at baseline ≤ 7 days prior to study enrollment and on day 1 of each cycle. CA125 response was defined as > 50% reduction confirmed after 28 days per the GCIG CA125 response criteria^12^. CA125 PD was defined as greater than two times the upper limit of normal if baseline was normal with PD confirmed after at least 7 days, in accordance with GCIG CA125 PD criteria^12^. If the baseline CA125 was elevated, PD was defined as greater than two times the upper limit of normal (if the CA125 nadir value was ≤ 16.2 units/mL) or doubling of nadir (if the CA125 nadir was > 16.2 units/mL).

### Statistical analysis

All statistical analysis was performed using R Statistical Software v.4.3.2 (2023-10-31 urct) and figures generated in Graphpad Prism version 10.2.0 for MacOS (GraphPad Software, Boston, Massachusetts, USA). The Kaplan–Meier method was used to estimate PFS and OS. Between subgroup differences in PFS and OS were assessed using cox proportional hazards models. Variables used in the multivariate analysis included GCIG CA125 response status, included platinum-sensitivity (platinum-sensitive vs. platinum-resistant), BRCA mutation status (BRCA1/2-mutated vs. BRCA-wildtype), previous PARP inhibitor therapy (yes vs. no), FIGO stage at diagnosis (FIGO stage III vs. FIGO stage IV), and ECOG performance status at study onset (ECOG status 0 vs. 1).

To assess the relationship of GCIG CA125 response with RECIST response, contingency tables were used to calculate the positive predictive value (PPV) and negative predictive value (NPV). PPV is defined as the probability that patients with CA125 response also had RECIST response (either complete response (CR) or partial response (PR) by RECIST v1.1 criteria)^13^. NPV defined as the probability that patients without CA125 response also did not have RECIST response (either stable disease (SD) or PD by RECIST v1.1)^13^, was also computed. Differences in overall response rate (ORR) between GCIG CA125 responders and GCIG CA125 non-responders were analyzed using Fisher’s exact test.

The relationship between GCIG CA125 PD and RECIST PD was also assessed for PPV and NPV. If GCIG CA125 PD occurred, patients were assessed to determine whether RECIST PD occurred within one cycle prior to or after GCIG CA125 PD. RECIST PD was included in PPV analysis as positive only if RECIST PD occurred within one cycle prior to after to GCIG CA125 PD. All 95% confidence intervals (95% CI) for PPV and NPV were estimated using the Epi: Statistical Analysis in Epidemiology R package (v2.27.1; 2022).

## Results

**Figure 1:**
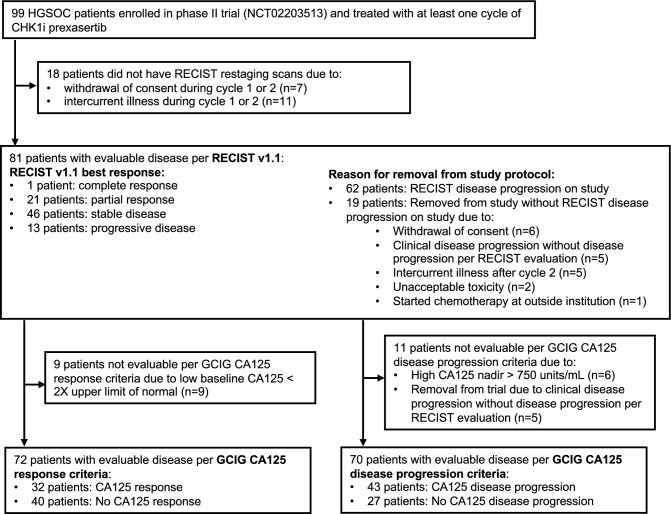
Study population of HGSOC patients treated with prexasertib. Flow chart shows patients with evaluable disease by RECIST v1.1 and GCIG CA125 criteria. *CA125* cancer antigen 125, *CHK1i* cell cycle checkpoint kinase 1 inhibitor, *GCIG* Gynecologic Cancer InterGroup, *HGSOC* high grade serous ovarian cancer, *RECIST* response evaluation criteria in solid tumors.

**Figure 2:**
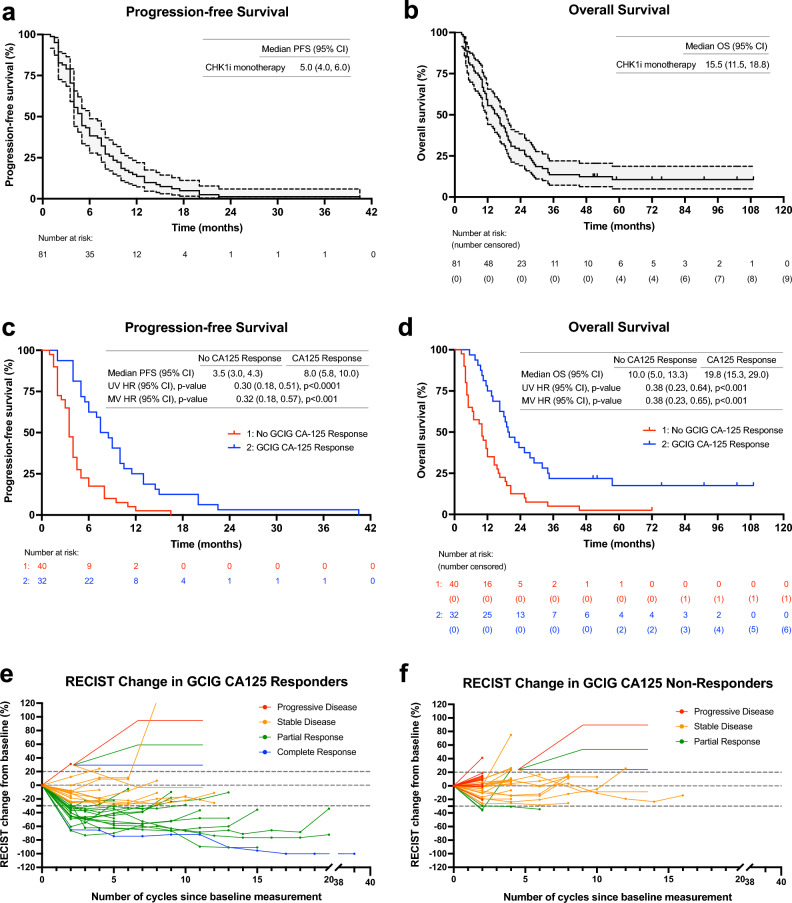
Kaplan–Meier curves of (**a**) PFS and (**b**) OS of all patients treated with prexasertib with evaluable disease by RECIST (95% confidence interval shaded in gray). Censored patients are denoted by tick marks along the survival curves. Kaplan–Meier curves showing (**c**) PFS and (**d**) OS in patients separated by Gynecologic Cancer Intergroup (GCIG) CA125 response or no response. Spider plot of RECIST tumor size change from baseline in (**e**) patients with GCIG CA125 response and (**f**) patients with no GCIG CA125 response. *CA125* cancer antigen 125, *CHK1i* cell cycle checkpoint kinase 1 inhibitor, *GCIG* Gynecologic Cancer InterGroup, *HR* hazard ratio, *MV* multivariate, *OS* overall survival, *PFS* progression-free survival, *RECIST* response evaluation criteria in solid tumors, *UV* univariate.

99 patients were enrolled and had baseline CA125 and CT scans. Of these, 18 patients were removed from the study prior to first restaging scans due to intercurrent illness during cycle 1 (n = 11) or withdrawal of consent during cycle 1 (n = 7). Thus, 81 patients had disease evaluable for response and PD by RECIST criteria (Fig. 1). The baseline characteristics of all 81 RECIST eligible participants are described in Table 1. Briefly, all patients were heavily pretreated with a median of 4 prior systemic therapies (interquartile range (IQR): 3 to 6). Most patients (68/81 [84.0%]) had platinum-resistant or platinum-refractory disease, and 18 (22.2%) patients had BRCA1/2 mutations. The median PFS was 5 months (95% CI: 4.0 to 6.0) (Fig. 2a), consistent with previous clinical studies of CHK1i therapy in HGSOC^6,8,14^. Notably, the median OS in this heavily pre-treated population was 15.5 months (95% CI: 11.5 to 18.8) (Fig. 2b). Across all patients with RECIST measurable disease, 1 patient had a CR lasting 39 months, and 21 patients had a PR, for an ORR (CR + PR) of 27.2% (22/81).

**Table 1:** Patient characteristics.

Female sex, N (%)	81 (100%)
Age in years, median (IQR)	63.2 (56.2, 68.4)
Prior number of systemic therapies, median (IQR)	4 (3, 6)
*Prior therapy exposure*	
Prior platinum-based therapy	81 (100%)
Prior PARP inhibitor	47 (58.0%)
*ECOG performance status at start of trial, N (%)*	
0	15 (18.5%)
1	66 (81.5%)
*FIGO stage at diagnosis, N (%)*	
I or II	3 (3.70%)
III	60 (74.1%)
IV	18 (22.2%)
*Race, N (%)*	
African American/Black	5 (6.17%)
Asian	2 (2.47%)
White	73 (90.1%)
Unknown	1 (1.23%)
*BRCA mutation status, N (%)*	
BRCA-mutant	18 (22.2%)
BRCA-wildtype	63 (77.8%)
*Platinum sensitivity, N (%)*	
Platinum-sensitive	13 (16.0%)
Platinum-resistant	67 (82.7%)
Platinum-refractory	1 (1.23%)
Baseline CA125, median (IQR)	349 (107, 1500)*
*Baseline CA125, N (%)*	
Normal (0–16.3 units/mL)	3 (3.70%)
Abnormal (> 16.3 units/mL)	78 (96.3%)

*ECOG* Eastern Cooperative Oncology Group, *FIGO* International Federation of Gynecology and Obstetrics, *IQR* interquartile range, *RECIST* response evaluation criteria in solid tumors. *Values > 1500 units/mL calculated as 1500 units/mL.

Patients evaluable by both RECIST criteria and GCIG CA125 criteria were included to compare outcomes of each criteria method. For GCIG CA125 response criteria, 72 of 81 RECIST evaluable patients were eligible (8 excluded due to a low CA125 value at baseline [less than 2X the upper limit of normal]) (Fig. 1). 32 had GCIG CA125 response (n = 32/72, 44.4%) and the remaining 40 patients did not have a GCIG CA125 response (n = 40/72, 55.6%). 17 patients had both RECIST and GCIG response (n = 17/72, 23.6%).

### Relationship between CA125 response criteria and clinical outcomes

**Figure 3:**
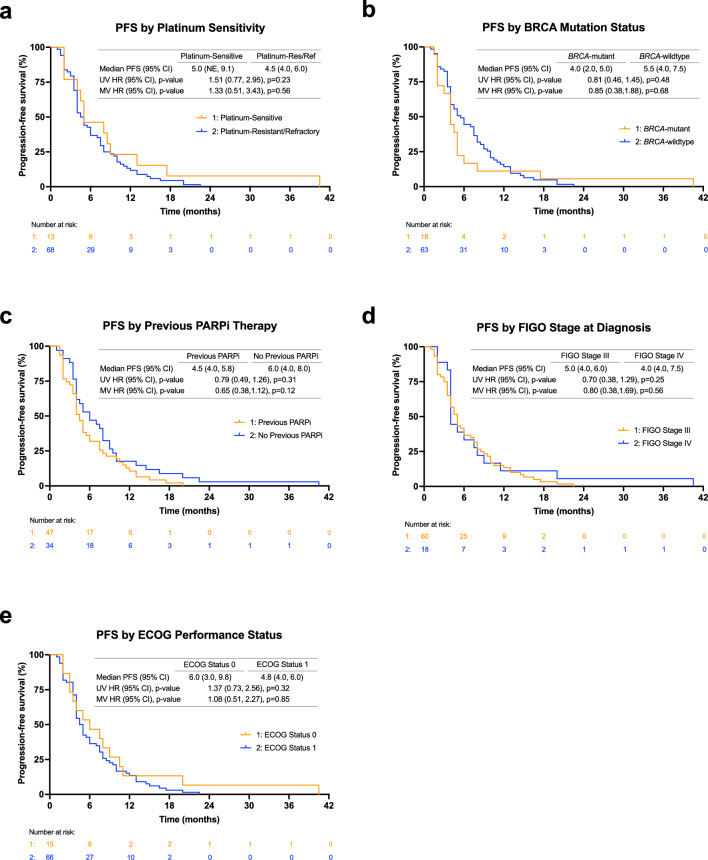
PFS by subgroups including (**a**) platinum sensitivity (**b**) BRCA mutation status, (**c**) previous PARP inhibitor (PARPi) therapy exposure, (**d**) FIGO stage at diagnosis, and (**e**) ECOG performance status at the start of therapy. *ECOG* Eastern Cooperative Oncology Group, *FIGO* International Federation of Gynecology and Obstetrics, *HR* hazard ratio, *MV* multivariate, *NE* not evaluable, *PARP* poly(ADP-ribose) polymerase, *PFS* progression-free survival, *UV* univariate.

**Figure 4:**
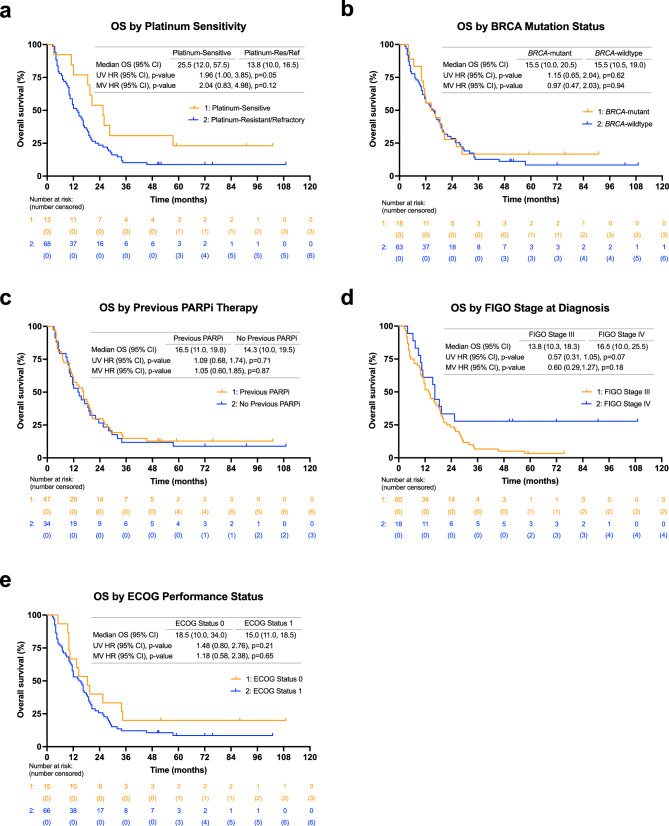
Overall survival by subgroups including (**a**) platinum sensitivity (**b**) BRCA mutation status, (**c**) previous PARP inhibitor (PARPi) therapy exposure, (**d**) FIGO stage at diagnosis, and (**e**) ECOG performance status at the start of therapy. *ECOG* Eastern Cooperative Oncology Group, *FIGO* International Federation of Gynecology and Obstetrics, *HR* hazard ratio, *MV* multivariate, *NE* not evaluable, *PARP* poly(ADP-ribose) polymerase, *OS* overall survival, *UV* univariate.

A higher ORR was associated with GCIG CA125 response (53.1%, 17/32), compared to those without GCIG CA125 response (7.5%, 3/40) (*p* < 0.0001). Additionally, CA125 responders were associated with a higher PFS at 8.0 months compared to CA125 non-responders at 3.5 months (hazard ratio [HR]: 0.30, 95% CI: 0.21–0.56, *p* < 0.0001) (Fig. 2c). Similarly, a higher OS correlated with CA125 responders at 19.8 months compared to CA125 non-responders at 10.0 months (HR: 0.38, 95% CI: 0.23–0.64, *p* < 0.001) (Fig. 2d). Multivariate (MV) analysis further demonstrated the relationship of CA125 response with improved PFS and OS (PFS MV HR: 0.32, 95% CI: 0.18–0.57, *p* < 0.001; OS MV HR: 0.38, 95% CI: 0.23–0.65, *p* < 0.001). In the post-hoc subgroup analysis, no significant differences were observed in PFS according to platinum-sensitivity, BRCA mutation status, previous PARP inhibitor therapy, FIGO stage at diagnosis, or ECOG performance status (Fig. 3). Likewise, no significant differences were observed in OS in these subgroups (Fig. 4), except for an increasing trend in OS of platinum-sensitive patients compared to platinum-resistant/refractory patients (median OS: 25.5 months vs. 13.8 months, univariate (UV) HR: 1.96, 95% CI: 1.00 to 3.85, *p* = 0.05) though this trend was not observed in the MV analysis (MV HR: 2.04, 95% CI: 0.83 to 4.98, *p* = 0.12). All values for UV and MV analysis are additionally shown in Supplementary Table 1.

Since the scans were scheduled every 2 cycles while CA125 was collected every cycle, we hypothesized that CA125 response may predict early the tumor response measured by RECIST. In patients with CA125 response, response was recorded after 1 cycle of therapy in 27/32 patients (median time to GCIG CA125 response: 34 days, IQR: 29.8 to 39.5). In all 21 patients with RECIST response, response was observed at the first restaging scans (after two cycles of therapy).

### Predictive value of CA125 response criteria for RECIST response

**Figure 5:**
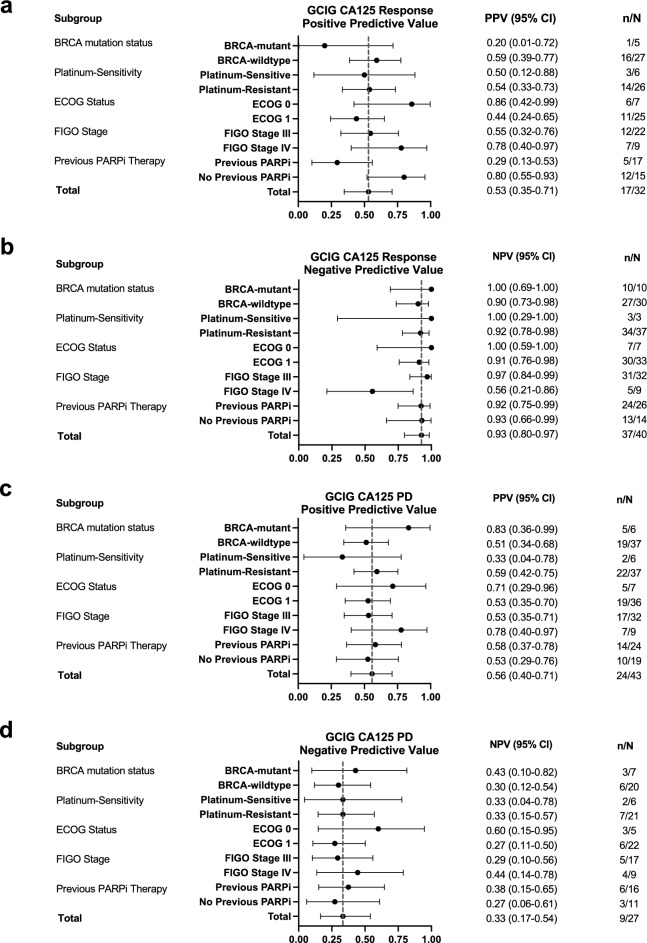
Forest plots showing (**a**) positive predictive values (PPV) and (**b**) negative predictive values (NPV) of Gynecologic Cancer Intergroup (GCIG) CA125 criteria response predicting RECIST response in patient subgroups. n/N refers to the number of (CA125 response and RECIST response)/([CA125 response and RECIST response] + [CA125 response and RECIST non-response]) for PPV and (CA125 non-response and RECIST non-response)/([CA125 non-response and RECIST non-response] + [CA125 non-response and RECIST response]) for NPV. Forest plots showing (**c**) PPV and (**d**) NPV of GCIG CA125 criteria disease progression (PD) predicting RECIST PD in patient subgroups. n/N refers to the number of (CA125 PD and RECIST PD)/([CA125 PD and RECIST PD] + [CA125 PD and RECIST non-PD]) for PPV and (CA125 non-PD and RECIST non-PD)/([CA125 non-PD and RECIST non-PD] + [CA125 non-PD and RECIST PD]) for NPV.

In patients evaluable by both RECIST and CA125 criteria (n = 72), approximately half of patients (n = 32) had a CA125 response. Of these, 17 also had RECIST response (Fig. 2e), resulting in a PPV of 53% (95% CI: 35 to 71%) (Table 2). Of note, while the 15 remaining patients did not meet the RECIST criteria for response, 7/15 had durable SD lasting longer than 6 months (Fig. 2e). Of those without CA125 response (n = 40), 3 had RECIST response and 37 did not (Fig. 2f), resulting in a NPV of 93% (95% CI: 80 to 98%) (Table 2). Additionally, the three patients without CA125 response, but with RECIST response did not achieve long-term responses: one patient had PR on the first staging scans at 2 months, then PD on the subsequent scans at 4 months, one patient also had PR on the first staging scans, then PD at 6 months, and one patient had PR but withdrew consent prior to cycle 4 despite improvement in tumor size (Fig. 2f). No significant differences were observed in PPV and NPV across all subgroups (Fig. 5a,b).

**Table 2:** Predictive value of GCIG CA125 response/no response.

	RECIST response	No RECIST response	Total	
GCIG CA125 response	17	15	32	**PPV: 53%** 95% CI: 35 to 71
No GCIG CA125 response	3	37	40	**NPV: 93%** 95% CI: 80 to 98
Total	20	52	72	

Positive predictive value (PPV) and negative predictive value (NPV) of GCIG CA125 response criteria to predict RECIST response (Best RECIST response of CR/PR) or no RECIST response (Best RECIST response of SD/PD). PPV: probability of patients with CA125 response that also had RECIST response, NPV: probability that those without CA125 response also did not have RECIST response.

*CA125* cancer antigen 125, *CI* confidence interval, *GCIG* Gynecologic Cancer Intergroup, *NPV* negative predictive value, *PPV* positive predictive value, *RECIST* response evaluation criteria in solid tumors.

Significant values are in bold.

### Association between CA125 PD and RECIST PD

GCIG CA125 PD was evaluated independently of GCIG CA125 response. For GCIG CA125 PD, 70 of 81 RECIST evaluable patients were evaluable (11 were excluded due to high CA125 nadir > 750 units/mL [n = 6] or clinical PD without progression per RECIST evaluation [n = 5]) (Fig. 1). Out of 70 patients evaluable by RECIST and CA125 PD criteria, 43 patients had CA125 PD (n = 43/70, 54.8%) and 24 of those had RECIST PD occurred within a month before or after CA125 PD (n = 24/70, 38.6%), resulting in a PPV of 56% (95% CI: 40 to 71%) (Table 3). Of the 27 patients without a CA125 PD, 9 patients also did not have a RECIST PD, resulting in a NPV of 33% (95% CI: 17 to 54%) (Table 3). PPV and NPV for PD were not different across the subgroups (Fig. 5c,d).

**Table 3:** Predictive value of GCIG CA125 PD/no-PD.

	RECIST PD	No RECIST PD	Total	
GCIG CA125 PD	24	19	43	**PPV: 56%** 95% CI: 40 to 71
No GCIG CA125 PD	18	9	27	**NPV: 33%** 95% CI: 17 to 54
Total	42	28	70	

Positive predictive value (PPV) and negative predictive value (NPV) of GCIG CA125 disease progression (PD) criteria to predict RECIST PD within one cycle of CA125 PD. PPV: probability of patients with CA125 PD also had RECIST PD, NPV: probability that those without CA125 PD also did not have RECIST PD.

*CA125* cancer antigen 125, *CI* confidence interval, *GCIG* Gynecologic Cancer Intergroup, *NPV* negative predictive value, *PD* disease progression, *PPV* positive predictive value, *RECIST* response evaluation criteria in solid tumors.

Significant values are in bold.

Lastly, 24 patients had PD by both CA125 PD criteria and RECIST evaluation, allowing for comparison of when PD occurred by each criteria method. The date of the initially observed CA125 PD tended to occur prior to the date of RECIST PD with a median of 28 days prior to RECIST response (IQR: 0, 87).

## Discussion

The clinical application of CA125 as a readily accessible and cost effective biomarker has been studied in predicting response for the chemotherapy in the adjuvant setting^9,15,16^. However, it is unclear how CA125 changes are reflected in the recurrent disease setting with small molecule inhibitors. In this study, we found that CA125 response was associated with a higher ORR and improved PFS and OS, however, had poor PPV in predicting RECIST response. Indeed, CA125 has a high NPV value for predicting RECIST response, in which patients without a GCIG CA125 response after the first cycle of prexasertib therapy are unlikely to have significant tumor reduction on imaging. It is noteworthy that both CA125 response and PD typically preceded changes on imaging, in part due to the increased frequency of CA125 evaluation compared to RECIST evaluation.

There are limited reports of the predictive value of CA125 response or progression when compared with RECIST tumor changes in relapsed ovarian cancers^13,17,18^. For instance, CA125 exhibited poor NPV for PD (33%, 95% CI: 29 to 38) in relapsed platinum-sensitive ovarian cancer patients receiving platinum-based combination therapy^13^. Similarly, CA125 exhibited poor NPV for PD (53%, 95% CI: 49 to 57) in a similar population receiving PARP inhibitor maintenance therapy^17^. Limited knowledge of CA125 use in newer therapies in the recurrent setting exists. The relationship between CA125 and tumor response has yet to been investigated in clinical studies with cell cycle checkpoint inhibitors^14,19,20^, and minimal data is available for other targeted therapies. Asad et al. has reported poor concordance between CA125 and RECIST response/PD for the combination of bevacizumab and sorafenib^18^. Additionally, in response to immune checkpoint inhibition, Boland et al. found that CA125 did not correlate well with tumor response. In their cohort, CA125 tended to increase regardless of whether the patient achieved clinical benefit (73% [11/15] in patients with clinical benefit ≥ 24 weeks vs. 82% [36/44] in patients without clinical benefit, *p* = 0.48)^21^ Our findings align with these observations, demonstrating discordance for CA125 in predicting PD during CHK1i prexasertib therapy though CA125 still shows potential for predicting no response to CHK1i.

It is also noteworthy that in real-world clinical practice, monitoring for PD often involves regular physical exams and measurements of CA125 without routine imaging^22^. Imaging is conducted only in suspected clinical recurrence due to cost and overall lack of data to support routine use, unlike clinical trials that require more frequent scans (e.g. every 2 months)^13,17,23^. Therefore, cases of recurrent disease where CA125 does not rise in concordance with tumor growth, may result in delayed diagnosis of PD. On the other hand, it is also important to note that any benefit from early detection of PD based on CA125 changes alone is debatable. The MRC OV05/EORTC 55,955 trial investigated early vs. delayed treatment based on CA125 PD in 1442 patients with ovarian cancer in remission with unelevated CA125 values after adjuvant platinum-based chemotherapy^24^. Interestingly, the authors found no difference in OS or quality of life outcomes, indicating that earlier treatment based on rising CA125 alone without clinical and radiographic changes does not necessarily lead to more favorable outcomes^24^.

Additionally, since 2011 when the definitions for GCIG CA125 response and PD were established^12^, new methods of CA125 mathematical modeling have been proposed and introduced into clinical practice^25,26^. These models have been investigated for platinum-based chemotherapy and PARP inhibition using large datasets, such as the CALPYSO, ARIEL2, and STUDY10 trials^25,27,28^. Nonetheless, mathematical models of CA125 are not yet available for many small molecule inhibitors, including CHK1i. As such, current use of CA125 in clinical practice for other targeted therapies should rely on readily available measures, such as GCIG CA125 criteria.

Early changes in peripheral blood biomarkers other than CA125, such as circulating tumor DNA (ctDNA), circulating tumor cells (CTCs) and immune cells, have been investigated in recurrent ovarian cancer as well, although are less accessible in clinical practice. Lee et al. reported ctDNA monitoring of minimal residual disease as a marker for predicting disease recurrence (PPV: 100%, NPV: 96.7%) in 27 patients on PARP inhibitor maintenance therapy^29,30^. Regarding CHK1i therapy specifically, Lampert et al. reported that an increase in the immunocompetence functional marker HLA-DR on total monocytes during the first 15 days of prexasertib treatment is associated with improved PFS (median PFS: 9.3 vs 3.5 months, *p* = 0.02)^31^. Furthermore, Giudice et al. showed that decreases in CTCs collected from peripheral blood samples expressing the epithelial markers EpCAM and MUC1 show improved PFS in recurrent HGSOC (median PFS: 7.5 vs. 4 months; HR: 0.41, 95% CI: 0.20–0.86, *p* = 0.02)^8^. However, use of these biomarkers requires further validation and they are not widely available for use in the community setting.

One major limitation of this study includes the small sample size for subgroup analysis. Specifically, low numbers in certain subgroups such as *BRCA*-mutated and platinum-sensitive patients (n < 20) are present due to the exploratory nature of this study and the trial design focused on heavily pretreated populations, which may have led to finding no differences between subgroups. Additionally, we were unable to account for the site of new or advancing lesions during PD. In Tjokrowidjaja et al. among participants with concordant CA125 and RECIST PD, a greater proportion had peritoneal disease as a site of recurrence (188/355, 53%) than among those with RECIST-only PD (164/454, 36%; *p* < 0.001)^17^. However, given the advanced disease in our study population, we were unable to classify patients based on site of disease during PD. A prospective study with a well-powered and more inclusive subgroup analysis would be beneficial to validate our findings.

## Conclusions

GCIG CA125 response is an independent predictor of response to the CHK1i prexasertib in HGSOC prior to the first restaging scans and is associated with a higher RECIST response rate and improved PFS and OS. GCIG CA125 response has a high NPV, indicating that patients without a CA125 response after the first cycle of CHK1i therapy are unlikely to see a reduction in tumor burden, which is additionally associated with worse PFS and OS. Therefore, these patients may require a change in treatment. However, GCIG CA125 response has poor PPV for RECIST response, indicating that it is uncertain whether patients with GCIG CA125 response will have response (CR or PR) on CHK1i treatment. When CA125 is used for monitoring progression of disease on CHK1i therapy, GCIG CA125 PD has poor PPV and NPV within a one cycle window, indicating CA125 cannot reliably detect RECIST PD while on CHK1i treatment. These findings need to be validated in large prospective studies.

### Supplementary Information

Supplementary Table 1.

## Data Availability

The datasets generated during and/or analyzed during the current study are available from the corresponding author on reasonable request.
