# Supplementary material for: Gynecologic Cancer InterGroup CA125 response has a high negative predictive value for CHK1 inhibitor RECIST response in recurrent ovarian cancer
Source: Sci Rep. 2024 Jul 29;14:17459. doi: 10.1038/s41598-024-68338-2 (PMC11286792; doi:10.1038/s41598-024-68338-2)
Supplement: Supplementary file 1 — Supplementary Table 1. [file 41598_2024_68338_MOESM1_ESM.docx]

| **Supplementary Table 1:** Univariate and multivariate cox proportional hazards models of prognostic factors associated with PFS and OS | | | | | | | | |
| --- | --- | --- | --- | --- | --- | --- | --- | --- |
|  | **Progression-free Survival** | | | | **Overall Survival** | | | |
|  | **Univariate** | | **Multivariate** | | **Univariate** | | **Multivariate** | |
| **Factor** | **HR**  **(95% CI)** | **p-value** | **HR**  **(95% CI)** | **p-value** | **HR**  **(95% CI)** | **p-value** | **HR**  **(95% CI)** | **p-value** |
| No GCIG CA125 Response vs. GCIG CA125 Response | 0.30  (0.18, 0.51) | p<0.0001 | 0.32  (0.18, 0.57) | p<0.001 | 0.38  (0.23, 0.64) | p<0.001 | 0.38  (0.23, 0.65) | p<0.001 |
| *BRCA*-mutated vs. *BRCA*-wildtype | 0.81  (0.46, 1.45) | p=0.48 | 0.85  (0.38, 1.88) | p=0.68 | 1.15  (0.65, 2.04) | p=0.62 | 0.97  (0.47, 2.03) | p=0.94 |
| Platinum-sensitive vs. Platinum-resistant/refractory | 1.51  (0.77, 2.95) | p=0.23 | 1.33  (0.51, 3.43) | p=0.56 | 1.96  (1.00, 3.85) | p=0.05 | 2.04  (0.83, 4.98) | p=0.12 |
| ECOG status 0 vs. ECOG status 1 at start of therapy | 1.37  (0.73, 2.56) | p=0.32 | 1.08  (0.51, 2.27) | p=0.85 | 1.48  (0.80, 2.76) | p=0.21 | 1.18  (0.58, 2.38) | p=0.65 |
| FIGO stage III vs. FIGO stage IV at time of diagnosis* | 0.70  (0.38, 1.29) | p=0.25 | 0.80  (0.38, 1.69) | p=0.56 | 0.57  (0.31, 1.05) | p=0.07 | 0.60  (0.29, 1.27) | p=0.18 |
| Previous PARP inhibitor therapy vs. no previous PARP inhibitor therapy | 0.79  (0.49, 1.26) | p=0.31 | 0.65  (0.38, 1.12) | p=0.12 | 1.09  (0.68, 1.74) | p=0.71 | 1.05  (0.60, 1.85) | p=0.87 |
| *Patients with FIGO stage I and II were excluded from analysis due to low n number (n<5).  **Abbreviations:** ECOG = Eastern Cooperative Oncology Group, FIGO = International Federation of Gynecology and Obstetrics, GCIG = Gynecologic Cancer Intergroup, PARP = poly(ADP-ribose) polymerase, PFS = progression-free survival, OS = overall survival | | | | | | | | |
